# Communicating statin evidence to support shared decision-making

**DOI:** 10.1186/s12875-016-0436-9

**Published:** 2016-04-06

**Authors:** Bruce Barrett, Jason Ricco, Margaret Wallace, David Kiefer, Dave Rakel

**Affiliations:** Department of Family Medicine and Community Health, University of Wisconsin-Madison, 1100 Delaplaine Court, Madison, WI 53715 USA; Department of Family Medicine and Community Health, University of Minnesota, Minneapolis, MN 55455 USA; HealthEast Care System, St Paul, MN 55102 USA; Group Health Cooperative, Madison, Wisconsin 53703 USA; Department of Family Medicine & Community Health, University of Wisconsin, Madison, WI 53715 USA

**Keywords:** Attitude to health, Cholesterol, Clinical significance, Cost-benefit analysis, Decision making, Evidence-based medicine, Guidelines, Lipids, Minimal important difference, Outcomes, Patient preference, Preventive cardiology, Primary care, Quality of life, Shared decision-making, Statins

## Abstract

**Background:**

The practice of clinical medicine rests on a foundation of ethical principles as well as scientific knowledge. Clinicians must artfully balance the principle of *beneficence*, doing what is best for patients, with *autonomy*, allowing patients to make their own well-informed health care decisions. The clinical communication process is complicated by varying degrees of confidence in scientific evidence regarding patient-oriented benefits, and by the fact that most medical options are associated with possible harms as well as potential benefits.

**Discussion:**

Evidence-based clinical guidelines often neglect patient-oriented issues involved with the thoughtful practice of shared decision-making, where individual values, goals, and preferences should be prioritized. Guidelines on the use of statin medications for preventing cardiovascular events are a case in point. Current guidelines endorse the use of statins for people whose 10-year risk of cardiovascular events is as low as 7.5 %. Previous guidelines set the 10-year risk benchmark at 20 %. Meta-analysis of randomized trials suggests that statins can reduce cardiovascular event rates by about 25 %, bringing 10-year risk from 7.5 to 5.6 %, for example, or from 20 to 15 %. Whether or not these benefits should justify the use of statins for individual patients depends on how those advantages are valued in comparison with disadvantages, such as side effect risks, and with inconveniences associated with taking a pill each day and visiting clinicians and laboratories regularly.

**Conclusions:**

Whether or not the overall benefit-harm balance justifies the use of a medication for an individual patient cannot be determined by a guidelines committee, a health care system, or even the attending physician. Instead, it is the individual patient who has a fundamental right to decide whether or not taking a drug is worthwhile. Researchers and professional organizations should endeavor to develop shared decision-making tools that provide up-to-date best evidence in easily understandable formats, so as to assist clinicians in helping their patients to make the decisions that are right for them.

## Background

The practice of medicine rests on foundations of knowledge accumulated over centuries, from simple observation to large and rigorous randomized controlled trials (RCTs). Following the principles of evidence-based medicine, systematic reviews of RCTs allow for authoritative interpretation of best available evidence. And yet, even with well-proven medical interventions, there are potential harms as well as benefits, which may be valued quite differently by individual patients. This, combined with varying levels of understanding among clinicians and patients, yields substantive complexity and uncertainty at the individual decision-making level. This paper uses the example of statins for preventing cardiovascular events (heart attacks and strokes, primarily), to discuss the principles and practice of evidence-informed shared decision-making, emphasizing the importance of individual values, and the effective communication of probabilities.

Cardiovascular (CV) disease, causing heart attack, stroke and other CV events, is the leading cause of death and disability in the developed world [1, 2]. Of the conventional risk factors, age, sex, and family history (genetic predisposition) are fixed, but blood pressure, cholesterol, blood sugar, tobacco use, stress, depression, diet, and exercise are all considered targets in the effort to reduce the impact of heart attack, stroke, and other patient-oriented CV outcomes.

For people with the CV risks of dyslipidemia, hypertension and diabetes, numerous pharmaceutical interventions are available. For people with moderate to severe hypertension, several classes of drugs appear to be effective in reducing stroke and heart attack risk [3, 4]. For mild hypertension, evidence of pharmaceutical effectiveness is less clear [5, 6]. Drugs aimed at diabetes can improve glycemic control, and may improve some microvascular outcomes, but have marginal effects on CV outcomes [7, 8]. While several types of cholesterol-targeting drugs have been shown to modify the lipid profile in favorable directions, only statins (HMG co-A reductase inhibitors) have been shown to reduce CV event rates [2, 9–11].

In the past several years, there has been a minor increase in evidence available regarding statins for preventing CV events, and a major change in the translation of evidence into guidelines [12–14]. The recent 2013 American Heart Association and American College of Cardiology (AHA/ACC) guidelines endorse statin treatment when 10 year CV event risk is as low as 7.5 %, [15] and the 2014 U.K. National Institute for Health and Care Excellence (NICE) guidelines suggest that clinicians “Offer atorvastatin 20 mg for the primary prevention of CVD to people who have a 10 % or greater 10-year risk of developing CVD” [2]. Only a few years earlier, guidelines from the same organizations supported statins only for people with 20 % or higher 10 year CVD event risk [16, 17].

This article focusses on statins for preventing CV events, but the principles of evidence-informed shared decision-making apply widely. See Table 1. The aims of this article are to:

**Table 1:** Main point summary

Clinical decision-making should be guided by patient values as well as best available evidence
Virtually all medical interventions have potential harms as well as potential benefits
Benefits and harms vary in terms of frequency, magnitude, impact, and importance to patients
Recently released guidelines endorse the use of statins to prevent cardiovascular (CV) events when the estimated 10 year CV event risk is as low as 7.5 %, a major change from previous guidelines, which endorsed preventive treatment when 10-year risk was 20 % or higher
Best evidence suggests that taking a statin pill every day for 10 years would reduce a 7.5 % risk by about 1.9 to 5.6 %. Similarly, a 20 % 10-year event risk could be reduced to 15 %.
Potential harms of statins are very low, but include myopathy, diabetes, and hepatotoxicity
Whether benefit harm trade-offs make a statin worthwhile is an individual patient decision
Practicing clinicians and health care delivery systems should strive to communicate best available evidence so that patients are able to make informed decisions about their health

1. Summarize current evidence regarding statins for preventing CV events
2. Identify limitations of the most recent guidelines
3. Outline an evidence-informed approach towards shared decision making
4. Use two case examples to illustrate this approach
5. Suggest a few potentially fruitful future directions for the development and use of clinical guidelines and decision aids

## Discussion

In order to frame our discussion of evidence regarding benefits, harms, uncertainty, and the complex task of shared decision-making, we present two hypothetical patients for your consideration. Please imagine that you practice general outpatient medicine and are familiar with the current guidelines.

Your first patient, Joe Smith, is a 45 year old non-smoking man with total cholesterol of 220 mg/dL and HDL of 30 mg/dL. He is taking chlorthalidone for hypertension, and his last blood pressure was 142/82 mmHg. According to the risk calculator available at http://cvdrisk.nhlbi.nih.gov [18] he has a 7 % chance of having a heart attack over the next 10 years, and hence may not be eligible for a statin according to the guideleines. However, he tells you that his heavy smoking father had a fatal heart attack at age 46, and that he wants to do everything he can to avoid a similar fate.

Your second patient, Mary Jones, also has a total cholesterol of 220 mg/dL and an HDL of 30 mg/dL. She is a 60 year old pack-a-day smoker with an untreated blood pressure of 154/88 mmHg. According to the same risk calculator, she has a 14 % 10-year risk of having a heart attack. As far as she knows, no one in her family has had a heart attack or stroke. She is reluctant to take medications, for reasons of cost and convenience, and also because several friends have had unacceptable side effects when taking blood pressure or cholesterol lowering medications.

### Benefits

Statins, also known as HMG co-A reductase inhibitors, have been studied extensively, and are considered proven effective medicines. More than two dozen large randomized controlled trials (RCTs) have enrolled more than 175,000 study participants [9–11, 19–21]. For perhaps two decades, statins have been considered beneficial in people who have already had a heart attack (secondary prevention). Over the past several years, there has been increasing consensus that statins used for primary prevention may reduce the rate of initial CV events, and related mortality, at least for those of sufficiently high risk [9, 22]. However, the impact of statins on all-cause mortality for people with dyslipidemia or other risk factors, but without previously known vascular disease, has been met with more controversy [9, 23]. Part of the controversy comes from the fact that many of the individual trials failed to demonstrate mortality or CV event reductions, perhaps because of inadequate statistical power. Pooling data through meta-analysis increases power, but brings limitations, including those related to diversity of populations sampled, outcomes assessed, statistical methods used, and specific medications and doses tested. It may be important to note that several of the so-called primary prevention trials included some people with known CV disease. Illustrating this point, a 2010 meta-analysis of 11 primary prevention trials (65,229 participants) included only those with high risk but no known CV disease, and failed to find statistically significant all-cause mortality reduction benefits [11]. However, the 2012 Cholesterol Treatment Trialists’ (CTT) meta-analysis of 22 trials (*n* = 134,537) [10] and the 2013 Cochrane report (18 trials; *n* = 56,934) [9] both found evidence of all-cause mortality benefit, but did include trials that had subjects with pre-existing CV disease.

Regardless of whether all-cause mortality benefits can be proven for moderate risk patients, it has become increasingly clear that statins can reduce CV events and associated mortality, and that these benefits appear to exist in moderate as well as high risk populations [9, 10, 21, 24]. The CTT and Cochrane reports strengthened consensus regarding the important benefits of statins, which led to the new NICE and AHA/ACC guidelines, which, if implemented widely, would dramatically increase the number of people taking statins [25].

Accepting that benefits have been well-proven, a logical next step is to look at magnitude, or degree of benefit. The 2012 CTT study focused on vascular event rates as a function of low density lipoprotein (LDL), and reported a 21 % relative risk reduction (RRR) per 1.0 mmol of LDL reduced (the average LDL reduction was 1.07 mmol/L) [10]. The 2013 Cochrane analysis reported an average RRR of 25 % for combined fatal and nonfatal CV events (95 % CI 0.19 to 0.30). Because actual risk varies as a function of baseline risk, this would translate to an absolute risk reduction of 5 % for a patient with a 20 % 10-year CV event risk, and 1.9 % for someone with a 7.5 % baseline risk. The Cochrane meta-analysis also reported statistically significant reductions in all-cause mortality, with relative risk reductions of approximately 14 %. Applying that to a high risk person with a 10-year chance of death of 10 % from any cause, taking a statin could reduce that risk to 8.6 %. For someone with more moderate risks, the chance of a CV event could be lowered from 10 to 7.5 %, for example, and the chance of death from any cause from a 3 to 2.6 %, over 10 years. Table 2 displays absolute risk reduction and number needed to treat benefits across a spectrum of baseline risks, using the 25 % CV event RRR suggested by the Cochrane analysts. We include 5-year as well as 10-year timespans as we feel the shorter timeline may be more interpretable for many patients.

**Table 2:** Chances of cardiovascular event with or without statin over 5-year and 10-year spans

5 % 10-year risk		7.5 % 10-year risk		10 % 10-year risk		15 % 10-year risk		20 % 10-year risk		30 % 10-year risk	
with statin	without statin	with statin	without statin	with statin	without statin	with statin	without statin	with statin	without statin	with statin	without statin
3.75 %	5 %	5.63 %	7.5 %	7.5 %	10 %	11.25 %	15 %	15 %	20 %	22.5 %	30 %
ARR = 1.25 %		ARR = 1.88 %		ARR = 2.5 %		ARR = 3.75 %		ARR = 5 %		ARR = 7.5 %	
NNT = 80		NNT = 53		NNT = 40		NNT = 27		NNT = 20		NNT = 13	
2.5 % 5-year risk		3.75 % 5-year risk		5 % 5-year risk		7.5 % 5-year risk		10 % 5-year risk		15 % 5-year risk	
with statin	without statin	with statin	without statin	with statin	without statin	with statin	without statin	with statin	without statin	with statin	without statin
1.88 %	2.5 %	2.81 %	3.75 %	3.75 %	5 %	5.63 %	7.5 %	7.5 %	10 %	11.25 %	15 %
ARR = 0.63 %		ARR = 0.94 %		ARR = 1.25 %		ARR = 1.88 %		ARR = 2.5 %		ARR =3.75 %	
NNT = 160		NNT = 107		NNT = 80		NNT = 53		NNT = 40		NNT = 27	

Based on 25 % relative risk reduction (Taylor, 2013)

*ARR* absolute risk reduction, *NNT* number needed to treat

### Harms

Statins are known to cause muscular pain and inflammation among some users [26–29]. The CTT collaboration estimates that the risk of serious myopathy attributable to statins is approximately 1 in 1000 users over a 10 year period [10]. Other studies put the risk much higher. For instance, a retrospective cohort study of 58,977 people reported a 19 to 26 % relative increase in musculoskeletal problems for those taking statins, corresponding to a number needed to harm (NNH) of 47 [30]. In another study of 107,835 people who had been prescribed a statin, some 57,292 discontinued, at least temporarily [31]. More than half of the 18,778 reported statin-related events were due to muscle pain or inflammation [31]. Data from the National Health and Nutrition Examination Survey (NHANES), with a large representative sample, reported a 50 % higher prevalence of muscle pain among statin users vs. non-users, corresponding to an NNH of 19 [32]. One trial randomized *n* = 1,016 people to pravastatin, simvastatin or placebo, and reported significant impacts on perceived energy and exertional fatigue in the statin groups [33]. Another small RCT among high-risk patients reported an almost complete blunting of laboratory-assessed cardiovascular fitness-enhancing effects of exercise, among those randomized to simvastatin, compared to placebo [34].

Statins are associated with increased incidence of diabetes [29, 35, 36]. A meta-analysis of 13 trials (91,140 participants) reported a 9 % relative risk increase of incident diabetes for those treated with a statin vs placebo, which translates to about 5 new cases of diabetes for every 1000 people taking a statin [35]. In the JUPITER trial, an overall 25 % increase rate of incident diabetes was reported in those randomized to rozuvastatin versus placebo, with substantially higher rates among women, suggesting about 28 new cases of diabetes per 1000 women per 5 years [37]. Additional diabetes risk data come from the Women’s Health Initiative study, which suggest a 48 % relative increase in new onset diabetes among women taking statins [38]. Statin-associated diabetes risks appear to be dose-dependent, with higher risks for those taking higher doses and high-intensity statins [39].

In addition to myopathy and diabetes, a wide array of statin-related side effects have been reported, but not conclusively demonstrated. These include hepatotoxicity, [29, 31, 40] cognitive impairment, [41] depression, [42] irritability, [43] osteoarthritis, [30] sexual dysfunction, [33] interstitial lung disease, [44] acute renal failure, [40] and cataracts [40]. These potential adverse effects have not been investigated rigorously, and different studies have reported different results [45–47]. While the evidence for statin-caused myopathy and diabetes is reasonably convincing, estimates of degree-of-risk are imprecise. For example, meta-analysis suggests that statins increase diabetes incidence by about 9 %, but the associated 95 % confidence intervals span a range from 2 to 17 %, [35] and, as noted above, the JUPITER trial testing a high-intensity statin reported even higher diabetes risk [37]. The European Atherosclerosis Society estimates the overall risk of developing statin-related muscle symptoms to be anywhere from 7 to 29 % [48]. Most of the other suspected adverse effects are even less well understood, and are better described as associations rather than attributable consequences [29]. A major limitation of the data regarding adverse effects derives from trial design. While great efforts are spent assessing potential benefits, the assessment of harms may be minimized or neglected. As a result, benefits are often understood with far greater confidence and precision than are harms.

### Uncertainty

There are several reasons why even the best RCTs and meta-analyses provide limited information regarding individual benefit and harm probabilities. For example, the validity of statistical inference depends on several assumptions, including data distribution normality and parameter independence, which are often not satisfied in RCT data sets. Selection bias is also important. Trial participants are rarely recruited using probability sampling, hence are not representative of general at-risk populations. This compromises both generalizability and relevance to individual patients. Outcome assessment and classification may be biased or limited, both by the design of data-capture methods, and by those analyzing, interpreting, and reporting outcomes. Blinding is often an issue, both for participants and trialists. For example, people randomized to placebo rather than statin will on average have higher LDL levels, which might influence decisions to stent or bypass coronary arteries. Revascularization procedures are often considered “major vascular outcomes” and are included along with heart attacks and stroke when assessing benefit rates, but may be influenced by unintentional unblinding. For example, using the published CTT data but excluding revascularization procedures, Abramson found that, “for people at low risk of cardiovascular disease (10 % risk over next 5 years), statins do not reduce the risk of death or serious illness” [49].

### Guidelines versus shared decision making

Historical luminaries such as Hippocrates and Osler exhorted physicians to place the patient’s interests above their own, and to consider patient values for medical decision-making. Nevertheless, only in recent decades has the medical profession begun to shift from a paternalistic “doctor knows best” stance towards one explicitly endorsing patient-centered, evidence-based, [50–52] shared decision-making [53–55]. The growing strength of patient-orientated shared decision-making in the U.S. is exemplified by the focus on patient-centered medical homes, [56, 57] the emphasis on patient-oriented evidence that matters (POEMs), [58–60] and the Patient-Centered Outcomes Research Institute (PCORI) [61]. In the U.K., the importance of patient-oriented decision-making is highlighted in the Equity and Excellence NHS white paper and in the National Health Service Constitution [62, 63]. Following these directives, the NICE lipid guidelines state, “Treatment and care should take into account individual needs and preferences. Patients should have the opportunity to make informed decisions about their care and treatment, in partnership with their healthcare professionals” [2].

Despite official endorsement of considering patient values, guideline committees often resort to a one-size-fits-all approach towards clinical decisions. For example, while the NICE guidelines leave open the possibility of individualized decision-making by saying, “***Offer*** atorvastatin 20 mg for the primary prevention of CVD to people who have a 10 % or greater 10-year risk of developing CVD,” the clinical decision for secondary prevention seems to have been made without the patient being consulted: “***Start*** statin treatment in people with CVD with atorvastatin 80 mg” [2] [Emphasis added]. The AHA/ACC guidelines acknowledge but undermine a patient-centered approach by stating, “The ultimate decision about care of a particular patient must be made by the healthcare provider and patient in light of the circumstances presented by that patient. As a result, situations might arise in which deviations from these guidelines may be appropriate” [15]. This wording implies that the recommended use of statins for people with 10-year CVD risks of ≥7.5 % depicted in the widely-reproduced decision tree should be considered as standard, and that a choice to not use a statin would be a deviation.

We are not the first to point out the tensions between shared decision-making and clinical guidelines. Recent editorials by Montori and colleagues discuss the risks of practice guidelines that neglect patient preferences, [51] and provide specific examples of how guidelines might not apply to individual patients [12]. Research can provide estimates of benefit and harm probabilities among populations, but even the best data cannot judge whether the estimated benefit-harm balance makes the intervention worthwhile for an individual patient. Using evidence-based guidelines alone for clinical decision-making violates the ethical principle of autonomy by removing patients’ values, goals and volition from the equation.

The increasing use of patient decision aids (PDAs) may be helpful in this regard, as there is good evidence that the use of PDAs may improve patient understanding, empower engagement and self-efficacy, and reduce decisional conflict [64]. For example, the National Heart Lung and Blood Institute [18] and the Mayo Clinic in the U.S., [65] NICE in the U.K. [66] and the arriba group in Germany [67] have developed PDAs using clear language and simple figures to portray potential benefits of statins for patients across various risk levels. The NHLBI PDA is very simple and easy for patients to use, but is limited in terms of risk factors included and CV outcomes predicted. The German arriba system provides a library of decision aids, perhaps helpful for busy clinicians wanting to use PDAs for a variety of clinical decisions [68]. The Mayo PDA allows for individualized risk estimation using any of three systems (AHA/ACC ASCVD, Framingham, Reynolds), and potential benefits in terms of two statin choices (standard vs. high intensity). Predicted benefits are based on 25 % (standard) or 40 % (high intensity) relative risk reductions in CV event rates. The NICE PDA predicts benefits based on 35 to 40 % relative risk reductions, and allows for individualized calculation of increased risk of diabetes from statin use. As very few of the trials in the CTT and Cochrane analyses reported CV event rate reductions of 30 % or greater, [10, 21] we feel that both the Mayo and the NICE PDAs may be overly optimistic, and that the 25 % RRR reported by the Cochrane group is more realistic for most patients.

While patient decision aids are an important step forward, the development and production of PDAs does not ensure appropriate use in clinic. Even the most well-designed PDAs are unlikely to be helpful if the clinician and patient do not have sufficient time, communication skills, or ability to understand benefit-harm probabilities (numeracy) [69, 70]. Guidelines are often used to set benchmarks for quality indicators, which are then used to judge performance of organizations and individual clinicians [71]. To us, it seems lamentable that clinicians are sometimes judged by guideline-adherence rather than ability to communicate evidence to assist patients in making the choices that are right for them. The neglect of shared decision-making in favor of seeking the highest possible level of one-size-fits-all “quality” metrics seems particularly misguided when one stops to think about the lack of evidence regarding what those goals should be. For example, to our best reading of the literature, there is no good evidence to help distinguish between the hypotheses that 80 % versus 20 % of the population with, say, a 15 % 10-year risk of a having a CV event, would want to take a statin for 10 years, if they truly understood the evidence regarding benefit and harm probabilities.

Guidelines provide benchmark cut-off points for decision-making. Benefit harm probabilities, on the other hand, naturally occur across continuums not easily reduced to yes/no decisional alternatives. Someone with an estimated 8 % 10-year risk may not have meaningfully higher chances of a heart attack than someone with a 7 % risk. And yet, the 2013 ACC/AHA guidelines promote statins in one case but not in the other. The same could be said of 9 % versus 11 % in regard to the NICE guidelines. Additional concerns arise from the risk calculators themselves, which are based on a variety of assumptions and data sets, and which yield a surprisingly wide variety of event risk estimations [72, 73]. Should treatment decisions change from one calculator to another, or when different blood pressure readings or LDL or HDL (high density lipoprotein) values from the same individual are used?

We believe that patient-centered guidelines regarding cholesterol treatment for CV event prevention should acknowledge the risk continuum, and should strive to avoid recommendations based on cut-off values. Future guidelines should strive to incorporate decision-aids and media tools to help illustrate the risk continuum across treatment choices. Expert panel recommendations should explicitly acknowledge that medical decisions should be based on the preferences and values of well-informed patients, and not on some committee-approved interpretation of randomized trial evidence. As such, guidelines may be framed as suggestions to guide shared decision-making, and not as directives for clinical care [51]. Physician performance and health care quality should not be judged based on the proportion of patients whose decisions fit within guideline recommendations.

### Pathways forward

For several decades, medical culture has been undergoing a gradual shift towards shared decision-making, where the physician’s expert knowledge is a necessary but insufficient ingredient when making medical decisions. The emergence and evolution of evidence-based medicine and shared decision-making are major leaps forward, as patients, clinicians, and health care delivery systems have an ever-improving set of tools and information to draw upon. Increased recognition of the value of the patient’s perspective and experiential knowledge is leading to a broader conception of the process and purpose of medical care [74, 75]. Nevertheless, central tenets of medicine, such as “first, do no harm” (Hippocrates) and “treat the patient, not the disease” (Osler) have changed little.

We suggest that the national organizations that develop treatment guidelines should pay more attention to potential harms, scientific uncertainties, and the clinical context in which their proclamations may be applied. Both national groups and local health care systems should develop patient-friendly written, tabular and graphical means of communicating evidence. Researchers should work to fill in knowledge gaps, especially concerning harms, where the evidence from pharma-funded clinical trials is woefully deficient.

One potentially fruitful research direction has to do with assessment of benefit harm tradeoff judgments. To improve on the concept of “minimal important difference” [76] (which neglects harms), we defined “sufficiently important difference” (SID) as “the smallest amount of patient-valued benefit that an intervention would require in order to justify associated costs, risks, and other harms” [77–80]. At the individual level, SID_i_ is the smallest amount of benefit that justifies the costs, inconveniences, risks and other harms associated with an intervention. The SID, also known as “smallest worthwhile effect,” [81, 82] is an evidence-informed judgment made by the person for whom the benefits or harms might apply. At the population level, the distribution of SID is largely unknown, but if assessed properly, could go a long way towards guideline improvement. For example, knowing the amount of benefit that would justify an intervention for 10, 50 and 90 % of the population (the SID_10_, SID_50_, SID_90_, respectively) could frame the range over which treatments might be recommended. For statin decision-making, the SID_i_ would be the magnitude of CV event reduction that would justify the increased risk of harms, costs and inconveniences associated with taking the pills. Although that research has yet to be done, we expect that SID_i_ values would vary widely across the at-risk population. People like Joe Smith with his estimated 7 % 10-year heart attack risk might feel that statin benefits outweighed harms, which others like Mary Jones with her 10-year 14 % risk might not.

## Conclusions

There is strong evidence that statins can reduce the risk of heart attacks and other cardiovascular events. The degree-of-benefit increases across the risk spectrum, so that people with higher risk derive greater benefit. For example, best evidence suggests that someone with a pre-existing 20 % 10-year risk of a CV event might reduce that risk by 5 to 15 %, while someone with a 7.5 % estimated risk might reduce that risk by 1.9 to 5.6 %. Probabilities of harms, such as inflammatory muscle pain and increased incidence of diabetes are known less precisely, but may be important to patients, and should be taken into consideration. Other patient-oriented factors, such as the positive value of reassurance that one-is-doing-all-that-one-can to prevent a heart attack, or negatively-valued attributes, such as co-pays or other monetary costs, or the hassle of having to take a pill every day, or the need to regularly see doctors and have blood drawn, may be important to patients, but have not been assessed properly, and are not addressed by current guidelines. Medicine’s guiding ethics, such as beneficence and autonomy, have been recognized since Hippocrates, and have in principle changed little since Osler’s days. What has changed substantively is the quality and quantity of evidence available, and the gradual shift from paternalism towards patient-orientation. We hope that this essay may beneficially contribute in this direction.

### Availability of data and materials

The analysis presented here is based on previously published data, as presented in the References section below.

## Abbreviations

ACC: American College of Cardiology
AHA: American Heart Association
ARR: absolute risk reduction
CTT: cholesterol treatment trialists
CV: cardiovascular
CVD: cardiovascular disease
HDL: high density lipoprotein
JUPITER: justification for the use of statins in prevention
LDL: low density lipoprotein
NHANES: National Health and Nutrition Examination Survey
NICE: National Institute for Health and Care Excellence
NNH: number needed to harm
NNT: number needed to treat
PCORI: Patient-Centered Outcomes Research Institute
POEMs: patient-oriented evidence that matters
QI: quality improvement
RCT: randomized controlled trial
RRR: relative risk reduction
SID: sufficiently important difference
